# A reliable and effective method of DNA isolation from old human blood paper cards

**DOI:** 10.1186/2193-1801-2-616

**Published:** 2013-11-19

**Authors:** Yang Song, Abrahim Fahs, Charles Feldman, Suraj Shah, Yali Gu, Yifan Wang, Roberto F Machado, Richard G Wunderink, Jiwang Chen

**Affiliations:** Institute for Genome Sciences, University of Maryland School of Medicine, Baltimore, MD 21201 USA; Section of Pulmonary, Critical Care, Sleep and Allergy, Department of Medicine, University of Illinois at Chicago, Chicago, IL 60612 USA; Department of Internal Medicine, Faculty of Health Sciences, University of the Witwatersrand, Johannesburg, South Africa; Department of Medicine, Northwestern University, Chicago, IL 60611 USA

**Keywords:** Blood paper card, DNA isolation, DN131, PCR, Caspase-12

## Abstract

Blood paper cards provide an effective DNA storage method. In this study, we used three DNA dissolving reagents (Tris-EDTA [TE] buffer, Tris–HCl buffer, and water) and one common commercially available kit (DN131 from MRC Inc) to elute DNA from 105 human blood paper cards collected up to 10 years ago. These DNA samples were used as templates for amplification of a single nucleotide polymorphism (SNP, C125T) region of human caspase-12 by PCR and a specific Taqman genotyping assay using the same amount of DNA. We show that DNA isolated by Tris–HCl buffer has higher yield and quality in comparison to DN131 solution. PCR success rate to amplify caspase-12 C125T SNP using Tris–HCl is comparable to the method using DN131 (89.5% vs 87.6%). The Taqman genotyping success rate using Tris–HCl is higher than using DN131 (81.9% vs 70.5%). Using TE or water, PCR success rates are lower than using DN131 (73.3% [TE]; 72.4% [H_2_O]), but Taqman genotyping success rates are comparable to the method using DN131 (70.5% [TE]; 79.1% [H_2_O]). We concluded that using Tris–HCl is a reliable and effective method to elute DNA from old human blood paper cards. The crude DNA isolated by Tris–HCl can be used to study genetic polymorphisms in human populations.

## Introduction

Blood paper cards are widely used in biomedical research to maintain human genomic DNA and blood pathogen DNA for genetic, pharmacogenetic and clinical testing. They provide an easier collection, transportation, and storage methods than other protocols (Dobbs et al. 2002; Mas et al. 2007; Soetens et al. 2008; Hardin et al. 2009; Ataei et al. 2011; Klassen et al. 2012; Klassen et al. 2013). Blood paper cards can be stored at room temperature under dry conditions with a desiccant, and do not need other special storage requirements. On average, blood paper cards can be stored ten years post-collection, ensuring that DNA can be accessed for future experimentation.

Currently, several kits are commercially available for DNA isolation from blood paper cards. These kits include GenSolve (Cat.# WB100050, BTC Private Limited) (Richard et al. 2011; Phyo et al. 2012), forensicGEM (Cat.# 95044-090, Zygem Corporation) (Moss et al. 2003), DNA IQ (Cat.# DC6700, Promega) (Barbaro et al. 2004; Rohland and Hofreiter 2007), and DNAzol (DN131, MRC Inc) (Chomczynski et al. 1997; Mackey et al. 1998; Chomczynski et al. 1998; Haak et al. 2009; Turner et al. 2011; Knowles et al. 2012). DN131 is a commonly used reagent for blood paper card DNA isolation because its protocol is straightforward, and the supernatant with blood paper card post centrifugation can be directly used for amplification of targeted genes by polymerase chain reaction (PCR). However, the volume of the supernatant cannot exceed 10% of the total PCR volume when DN131 kit is utilized. In addition, DNA isolated by DN131 includes excess proteins, which will further dampen efficiency of PCR amplification. Previously our team had used Tris–HCl (10 mM, pH7.4) and achieved a high success rate for mouse genotyping (Sysol et al. 2013). We therefore hypothesized that DNA dissolving solutions including Tris–HCl, TE or water may be feasible for blood paper card DNA isolation.

The aim of this study was to investigate the feasibility and effectiveness using Tris–HCl (10 mM, pH7.4), Tris-EDTA buffer (TE, 10 mM, pH7.6), water and DN131 to isolate DNA from 105 human blood paper cards. In addition, we examined whether the crude DNA isolated by these solutions was sufficient to amplify caspase-12 C125T SNP (rs#497116). To our knowledge, this is the first comprehensive study to compare the effectiveness and efficiency using these easily obtained laboratory solutions for blood paper card DNA isolation.

## Materials and methods

### Reagents

Reagents were from Sigma (St Louis, MO, USA), unless otherwise stated. The four liquid solutions used for DNA isolation from blood paper cards were: Tris–HCl (10 mM, pH7.4), Tris-EDTA (TE) buffer (10 mM, pH7.6), sterilized water, DN131 (Molecular Research Center, InC).

### Human blood paper cards

105 human blood paper cards were collected in South Africa up to 10 years ago using Schleicher & Schuell (S & S) 903 paper cards (Whatman Ltd). S & S 903 is a FDA approved medical device recommended by the International Screening Community for Neonatal Screening worldwide. This blood paper card is also widely used for the studies of personalized medicine, with its paper manipulated for strictly quality-controlled for serum uptake, absorption characteristics and lot-to-lot consistency, ensuring reproducibility of results. Each blood paper card has been well separated by a sterilized plastic bag. Five circular blood samples were located in each blood paper card, and each blood circle contained a blood volume of approximately 75 μl. Endorsement to use these human blood paper cards has been permitted by the Northwestern University Institutional Review Board.

### DNA isolation

Outlined in Table 1 is the DNA isolation procedure utilized for this study. Four blood circles from each sample were used and cut into small pieces using a sterilized scissor and forceps. The small pieces from each blood circle were distributed into a 1.5 ml sterilized Eppendorf tube. As a result, four blood circles from one human sample were placed into four Eppendorf tubes, each containing one of the four different DNA dissolving solutions mentioned as above. The volume of each reagent used was 500 μl, ensuring that the entire blood paper card pieces were immersed within the reagent. Tubes were then placed in a rotator overnight at 4°C. After an overnight rotation, samples were heated at 95°C for 10 min, and then centrifuged at room temperature for 1 minute at 10,000 g. The supernatant (approximately 480 μl) was transferred into a new 1.5-ml Eppendorf tube. DNA quantity and quality measured using ND-1000 Spectrophotometer (Nano-Drop technologies, Inc). DNA quality was evaluated by the ratios of A260/A280 and A260/A230 (absorption values at the different wavelengths).

**Table 1:** **Procedure for DNA isolation from blood paper card**

Step	Procedure
1	Cut a blood paper card spot to 4 small pieces of equal size
2	Transfer paper card pieces to an eppendorf tube with an isolation solution
3	Rotate the tubes overnight at 4°C
4	Heat the tubes at 95°C for 10 min
5	Centrifuge at 10,000 g at the room temperature for 10 min
	The supernatant can be used directly for PCR

### PCR analysis

The supernatant obtained as above was directly used for amplification of a SNP (rs#497116) in human caspase-12 by PCR. The sequences of the two primers we used are: forward primer, 5′ GTCATTCTGTGTGTATTAATTGC3′; reverse primer, 5′ CCTATAATATCATACATCTTGCTC3′. Two different PCR amplification systems were used. System 1: 25 μl of Platinum^®^ PCR SuperMix (Cat.# 11306-016, Life Technologies Inc), 0.5 μl of mixed forward and reverse primers (10 μM) with a certain amount of crude DNA solution in a final reaction volume of 28 μl. System 2: using GoTaq DNA polymerase (Cat.# M3005, Promega Corporation). We added 5 μl of 5 × PCR buffer, 0.5 μl of mixed forward and reverse primers (10 μM), 0.3 μl Taq enzyme (5 units/μl), 1 μl dNTP (10 mM), 0.6 μl 25 mM MgCl_2_ with a certain amount of crude DNA solution in a final reaction volume of 25 μl. In system 1, the same amount of DNA was used for each sample according to 2 μl DNA amount isolated by DN131; in system 2, we used 2 μl DNA isolated by DN131, but with 16 μl DNA from the other three DNA isolation solutions. In both systems, the DNA volume from DN131 group we used (2 μl) was approximately 10% of the total PCR volume according to the manufacturer’s instruction. Cycling conditions were 94°C 2 min, 55°C 40 s, 72°C 1.5 min, two cycles → 94°C 20 s, 55°C 40 s, 72°C 1 min, 40 cycles → 72°C 8 min. Agarose-gel electrophoresis (2%) and ethidum bromide staining were used to check the PCR product, whose size is approximately 314 bp.

### Taqman genotyping assay

A Taqman genotyping assay [Applied Biosystems Inc (ABI), Foster City, CA] was also used to genotype the caspase-12 C125T SNP using the same amount of DNA isolated from the four solutions mentioned as above. The specific genotyping assay ID number for caspase-12 C125T is C_2411553_20. The genotyping was conducted in a 384-well plate with a total volume 10 μl in a C1000™ Thermal Cycler (Bio-Rad). The DNA amount we used is according to the DNA amount in 1 μl from DN131 isolated DNA samples.

### Data analysis

All the data were reported as mean and standard error (SEM). DNA concentration and DNA quality data analysis were performed on average of two repeated measurements. Statistical analysis was performed using GraphPad Prism 5.1 (GraphPad Software, La Jolla, CA). Normality of data distribution was assessed by using the Kolmogorov-Smirnov test. Because only DNA concentrations and A260/A230 ratios from the four groups were normally distributed, *T*-test was used to evaluate their differences between methods. Wilcoxon Rank-Sum test was used to examine the A260/A280 differences between methods including DN131. The agreement between DN131 and others (Tris–HCl, TE, H_2_O) was assessed by Bland-Altman plots using BA.plot package in R (Myles and Cui 2007). The differences between DN131 and our methods (Tris–HCl, TE, H_2_O) were plotted against the average values ([DN131 + H_2_O (TE or Tris–HCl)]/2) respectively. 95% prediction intervals (limits of agreement) of differences and Pearson correlation for “line of agreement” are also reported. The mean difference (Tris–HCl [TE or H_2_O] – DN131) was defined by a solid horizontal line. Two dashed lines represent the two standard deviations (SD) above and below the mean difference line, which correspond to the limits of agreement. Line of agreement was plotted by considering linear relation between the difference and mean. *p* < 0.05 was considered statistically significantly different.

## Results

### Quantity and quality of isolated DNA

**Figure 1:**
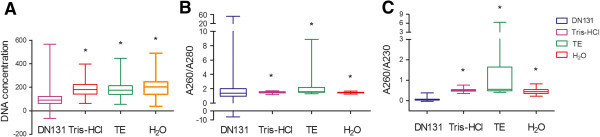
**Concentrations, A260/A280 and A260/A230 ratios of DNA isolated from old human blood paper cards with the four different reagents (DN131, Tris–HCl, TE, H**_**2**_ **O). A**. DNA concentrations (ng/μl); **B**. A260/A280 ratios; **C**. A260/A230 ratios. *, *p* < 0.05, compared to DN131 group. *T*-test was performed for DNA concentrations and A260/A230 ratios because these data are normally distributed, while A260/A280 data are not normally distributed. Wilcoxon Rank-Sum test was used to examine the A260/A280 differences between methods including DN131.

**Figure 2:**
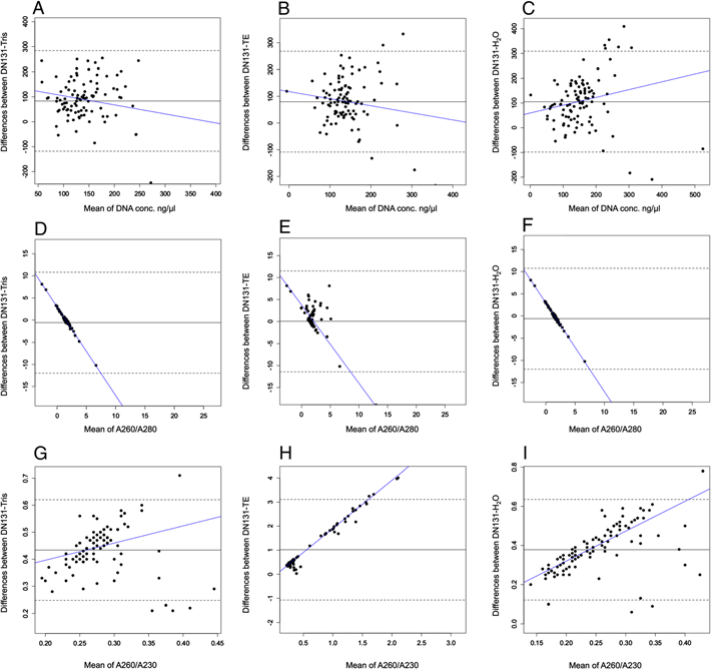
**Bland-Altman Plots of DNA concentrations, A260/A280 and A260/A230 ratios between the two methods using DN131 and using our solutions (Tris–HCl, TE or water). A**. Bland-Altman Plot of DNA concentrations between DN131 and Tris–HCl. Correlation R = 0.38 (*p* < 0.0001), slope = − 0.76 (*p* < 0.001), intercept = 193.27 (*p* < 0.0001); **B**. Bland-Altman Plot of DNA concentrations between DN131 and TE. Correlation R = 0.28 (*p* < 0.004), slope = − 0.46 (*p* = 0.004), intercept = 145.59 (*p* < 0.0001); **C**. Bland-Altman Plot of DNA concentrations between DN131 and H_2_O. Correlation R = 0.05 (*p* = 0.55), slope = 0.32 (*p* = 0.96), intercept = 59.65 (*p* = 0.99); **D**. Bland-Altman Plot of A260/A280 ratios between DN131 and Tris–HCl. Correlation R = 0.99 (*p* < 0.0001), slope = − 2.0 (*p* < 0.0001), intercept = 2.95 (*p* < 0.0001); **E**. Bland-Altman Plot of A260/A280 ratios between DN131 and TE. Correlation R = 0.97 (*p* < 0.0001), slope = − 1.97 (*p* < 0.0001), intercept = 3.51 (*p* < 0.0001); **F**. Bland-Altman Plot of A260/A280 ratios between DN131 and H_2_O. Correlation R = 0.99 (*p* < 0.0001), slope = −20 (*p* < 0.0001), intercept = 2.91 (*p* < 0.0001); **G**. Bland-Altman Plot of A260/A230 ratios between DN131 and Tris–HCl. Correlation R = 0.06 (*p* = 0.57), slope = 0.64 (*p* = 0.99), intercept = 0.27 (*p* = 0.99); **H**. Bland-Altman Plot of A260/A230 ratios between DN131 and TE. Correlation R = 0.99 (*p* < 0.0001), slope = 1.99 (*p* < 0.0001), intercept = −0.12 (*p* = 0.007); **I**. Bland-Altman Plot of A260/A230 ratios between DN131 and H_2_O. Correlation R = 0.47 (*p* < 0.0001), slope = 0.97 (*p* < 0.0001), intercept = 0.13 (*p* = 0.007).

DNA from 105 human blood paper cards was successfully recovered. Quantity and quality of these isolated DNA samples are shown in Table 2 and Figures 1-2. Using the same size of blood paper cards, concentrations of DNA recovered by Tris–HCl, TE, water and DN131 buffer were 190.0 ± 6.1, 183.5 ± 6.4, 203.5 ± 9.0 and 103.3 ± 8.6 ng/ml, respectively. Significantly higher DNA yield was obtained using Tris–HCl, TE, and water than using DN131 (*p* < 0.05, Figure 1A). Minimum and maximum values of DNA concentrations in four different groups were shown in Table 2, in which the variation between minimum and maximum values was greater in DN131 group than the other groups.

**Table 2:** **Quantity and quality of DNA isolated from old human blood paper cards using different reagents**

	DNA quantity	DNA quality	
Methods	(ng/ml)	A260/A280	A260/A230
	(min, max)	(min, max)	(min, max)
DN 131	103.3 ± 8.6	2.07 ± 0.6	0.06 ± 0.01
	(−64.9, 566.9)	(−6.64, 52.18)	(−0.03, 0.37)
Tris–HCl	190.0 ± 6.1	1.5 ± 0.01	0.5 ± 0.01
	(62.7, 397.7)	(1.17, 1.72)	(0.35, 0.75)
TE	183.5 ± 6.4	2.08 ± 0.1	1.06 ± 0.1
	(54.2, 444.8)	(1.30, 8.90)	(0.4, 6.25)
H_2_O	203.5 ± 9.0	1.5 ± 0.01	0.4 ± 0.01
	(38.8, 489.2)	(1.23, 1.68)	(0.22, 0.82)

In each space, values are represented as mean ± standard error. The minimum (min) and maximum (max) values are shown in parenthesis.

DNA quality was assessed by A260/A280 and A260/A230. The mean ± standard errors of these ratios with their minimum and maximum values were shown in Table 2 and Figure 1. According to Wilcoxon Rank-Sum test of the A260/A280 differences between methods, Tris–HCl, TE and H2O groups were significantly different from DN131 group (*p* < 0.05). In terms of A260/A230 ratio, DNA isolated by DN131 had the lowest values (0.06 ± 0.01, *p* < 0.05), compared to the ones by Tris–HCl (0.5 ± 0.01), TE (1.06 ± 0.1) or water (0.4 ± 0.01). In terms of both A260/A280 and A260/A230 ratio differences between the minimum and maximum values, DN131 group was greater than the other three groups, and TE group was higher than Tris–HCl or H_2_O group (Table 2).

According to the Bland-Altman plots (Figure 2), most of the points lie within the 95% limits of agreement. The mean DNA concentration difference between H_2_O and DN131 was 105.21 ng/μl, while the limit of agreement was −98.32 to 308.74. Most of the spots (96 out of 104) were within the limit of agreement. The slope of line of agreement almost parallel to the axis of average of H_2_O and DN131 (*p* = 0.96, R = 0.05), which indicates the concordance between H_2_O and DN131 was independent of the two methods. The mean DNA concentration difference between the TE and DN131 was 79.72 ng/μl, while the limit of agreement was −108.64 to 268.07. Most of the spots (99 out of 104) were within the limit of agreement. The slope of line of agreement was not parallel to the axis of average of the TE and DN131 (*p* = 0.004, R = 0.28). The mean DNA concentration difference between the Tris–HCl and DN131 was 83 ng/μl, while the limit of agreement was −118.11 to 284.13. Most of the spots (99 out of 104) were within the limit of agreement. The slope of line of agreement was not parallel to the axis of average of the Tris-HCl and DN131 (*p* < 0.0001, R = 0.38). Agreements were consistently high at TE and Tris–HCl, which further supports the significantly higher DNA yield obtained using Tris–HCl or TE than using DN131 based on *T*-test. In addition, the mean differences in the A260/280 ratios between DN131 and Tris–HCl, TE or H_2_O were −0.58 (from −11.97 to 10.8, limit of agreement), 0.035 (from −11.42 to 11.5) and −0.61 (from −11.98 to 10.76), respectively. These mean differences of the A260/A280 ratios combined their slope p values (all < 0.0001) indicates that there is significant difference in the DNA quality assessed by the A260/A280 ratios between methods including DN131, which is consistent with our Wilcoxon Rank-Sum test (Figure 1B). In addition, the mean differences in the A260/230 ratios between DN131 and Tris–HCl, TE or H_2_O were 0.43 (from 0.25 to 0.62, limit of agreement), 1.02 (from −1.07 to 3.11) and 0.37 (from 0.12 to 0.64), respectively. All the agreements were consistently high at Tris–HCl, TE and H_2_O, which further confirms the DNA quality evaluated by A260/A230 in Tris–HCl, TE or H_2_O is better than DN131 group based on *T*-test (Figure 1C).

### PCR amplification and Taqman genotyping assay of caspase-12 C125T SNP

**Figure 3:**
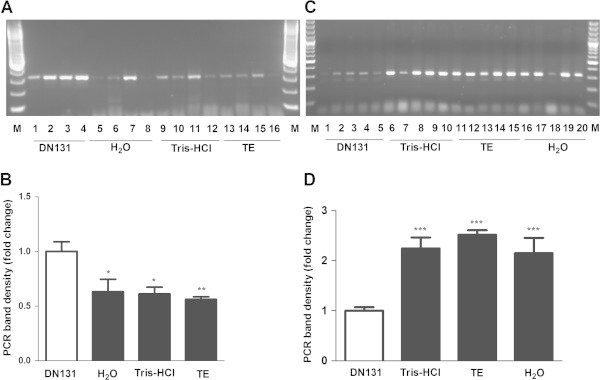
**PCR amplification of a DNA fragment of caspase-12 gene covering C125T SNP (rs497116) using 105 DNA samples isolated by different reagents (DN131, Tris–HCl, TE and water) with two different PCR amplification systems. A**. A representative image of PCR amplified bands separated by a 2% agarose gel electrophoresis for system 1, which was detailed in Materials and methods. PCR bands were visualized under UV light after ethidium bromide staining; **B**. PCR band density quantification by Image J for Figure 3A. *, *p* < 0.05; **, *p* < 0.01, compared to DN131; **C**. A representative image of PCR amplified bands separated by a 2% agarose gel electrophoresis for system 2, which was detailed in Materials and methods; **D**. PCR band density quantification by Image J for Figure 3C. ***, *p* < 0.001, compared to DN131.

PCR results using DNAs isolated by the four different reagents with two different PCR systems were analyzed and compared using agarose gel electrophoresis. A representative image of PCR-amplified caspase-12 C125T SNP fragment was demonstrated for system 1(Figure 3A) and system 2 (Figure 3C), respectively. The PCR-amplified band density was quantified and shown in Figure 3B for system 1 and in Figure 3D for system 2. As shown in Table 3, using the same amount of DNA template (system 1), PCR success rate is highest for Tris–HCl (89.5%, 94/105), with 87.6% (92/105) for DN131, 73.3% (77/105) for TE and 72.4% (76/105) for H_2_O. However PCR band density in DN131 group is significantly higher than the other three groups (Figure 3B, p < 0.05). When volume of DNA samples isolated by Tris–HCl, TE and water was increased to 16 μl (system 2), PCR amplification success rates were increased to 96.1% (99/103), 88.5% (92/105) and 78.1% (82/105), respectively. Meantime, PCR band density values in Tris–HCl, TE and water groups are all significantly higher than DN131 group (Figure 3D, *p* < 0.001).

**Table 3:** **Success rates of amplifying caspase-12 C125T SNP (rs497116) by PCR and a Taqman genotyping assay using four different reagents**

	PCR successful rate	PCR successful rate	Taqman genotyping successful rate
	(system 1)	(system 2)	
Method	(success#/total) (%)	(success#/total) (%)	(success#/total) (%)
DN131	(92/105) (87.6)	(75/105) (71.4)	(74/105) (70.5)
Tris–HCl	(94/105) (89.5)	(99/105) (96.1)	(86/105) (81.9)
TE	(77/105) (73.3)	(92/105) (88.5)	(74/105) (70.5)
H_2_O	(76/105) (72.4)	(82/105) (78.1)	(79/105) (79.1)

System 1 and system 2 are two different PCR amplification systems we used and described in Materials and methods.

When the same amount of DNA was used, success rates for Taqman genotyping of caspase-12 C125T SNP were shown in Table 3. Genotyping success rate for Tris–HCl (86/103; 81.9%) was the most successful, second was for water (79/105; 79.1%). DN131 or TE (both 74/105–70.5%) was the least successful.

## Discussion

In this study, we investigated for the first time about the feasibility of using three common DNA dissolving solutions (water, TE buffer, and Tris–HCl) to isolate DNA from old human blood paper cards. The quality and quantity of DNA samples isolated by the three common laboratory solutions are significantly higher than using DN131. The crude DNA isolation procedure minimizes both cost and preparation time, and can be completed in a single microcentrifuge tube in less than 10 min for a single blood card paper. Considering the downstream PCR amplification success rates, the crude DNA extraction using Tris–HCl without subsequent purification, was comparably successful to a commonly used, commercially available DNA isolation reagent, DN131. The PCR-amplified caspase-12 C125T SNP region can be further used for genotyping via DNA sequencing, restriction fragment length polymorphism and other approaches. Furthermore, the crude DNA isolated using Tris–HCl can be used for large-scale genotyping by Taqman genotyping system because Taqman genotyping success rates using Tris–HCl isolated DNA were higher than using DNA isolated by DN131, TE or water. Previously we also showed that Tris–HCl is a reliable and cost-effective DNA-isolation solution for a mouse genotyping using tails (Sysol et al. 2013).

DNA quality is generally evaluated by the A260/A280 ratio. A ratio of approximately 1.8 is commonly accepted as pure DNA (Wilfinger et al. 1997). If the ratio is lower than 1.8, it may indicate the presence of protein, phenol or other contaminants which absorb highly at or near 280 nm. In addition, pH values of DNA solutions will lead to variation of the A260/A280 ratios. In general, acidic solutions will under-represent the 260/280 ratio by 0.2–0.3, while a basic solution will over-represent the ratio by 0.2–0.3 (Wilfinger et al. 1997). In this study, DN131 is an alkaline solution (pH11.0) containing polyethylene glycol and other additives, and pH of the TE solution used is 7.6. This may explain why A260/A280 ratios are relatively higher in DN131 and TE groups than in Tris–HCl (pH7.4) and water groups. Why does the biggest standard error of A260/A280 ratios occur in DN131 group (2.07 ± 0.6) with the biggest difference between the minimum and maximum values (−6.64, 52.18, Table 2)? It may be attributed to its higher pH values and the existence of polyethylene glycol and other additives, which may more efficiently extract degraded RNA than other reagents. In general, degraded RNA has higher absorbance at 260 nm due to its higher ratio of Uracil compared to that of Thymine (Leninger 1975). In addition, DNA quality was also assessed by the A260/A230 ratio. The A260/A230 ratios for DNA are generally in the range of 2.0–2.2 and higher than the respective A260/A280 ratios (Wilfinger et al. 1997). If the ratio is lower than expected, it may suggest the presence of contaminants. DNA isolated by DN131 has lowest A260/A230 ratios, which indicate that more contaminants such as carbohydrates, polyethylene glycol and other additives in DN131 reagent which may have high absorbance near 230 nm. To accurately measure quality and quantity of isolated DNA, fluorescence-based DNA measurement is warranted in our future studies.

There are some limitations of this study. First, we used scissors and forceps for blood card cut, which is tedious and not suitable for large-scale studies. A paper puncher can be used to punch out the same size of blood card pieces which is suitable for large-scale studies. Punchers or other tools have to be well cleaned between samples to avoid any sample contaminations. Second, the other commercially available reagents and automatic DNA isolation system were not tested or compared. For example, Soetens et al. (2008) reported automatic easyMAG method which requires a NucliSens easyMAG automated extraction platform (BioMerieux, Boxtel, the Netherlands). This automatic DNA isolation system is faster and better controlled than the manual methods, and thus suitable for large-scale studies. The manual method we used is suitable for small number of blood card samples. Third, we only tested amplification of caspase-12 C125T SNP region, and did not try other genomic regions or blood pathogens. Therefore, in future studies, test of different genomic DNA regions and some blood pathogens by PCR or other methods is warranted.

In summary, this study demonstrates the efficacy of using a crude DNA isolation method from old human blood cards. Considering high success rates of PCR amplification and Taqman genotyping, and also availability in a common research laboratory, Tris–HCl is an alternative and reliable choice for DNA isolation from old blood paper cards. To our knowledge, this is the first systematic study to compare the reliability of this method compared to that of a commercially available reagent.
